# The Technology of Homogeneous Scar Tissue Creating as a Result of Ablation of the Atrial Wall with a Radiofrequency Bipolar Clamp: an Experimental and Clinical Study

**DOI:** 10.21470/1678-9741-2022-0274

**Published:** 2023

**Authors:** Sergey Alekseevich Vachev, Fedor Georgievich Zabozlaev, Sergey Vladimirovich Voronin, Ekaterina Alekseevna Chernavina, Aleksandr Vital’evich Troitskii

**Affiliations:** 1 Department of Cardiac Surgery, Federal Research and Clinical Center of Specialized Medical Care and Medical Technologies, Federal Medical and Biological Agency of the Russian Federation, Moskva, Russia; 2 Department of Pathological Anatomy, Federal Research and Clinical Center of Specialized Medical Care and Medical Technologies, Federal Medical and Biological Agency of the Russian Federation, Moskva, Russia; 3 Department of Anesthesiology, Federal Research and Clinical Center of Specialized Medical Care and Medical Technologies, Federal Medical and Biological Agency of the Russian Federation, Moskva, Russia

**Keywords:** Ablation, Atrial Fibrillation, Hear Atria, Hemorrhage, Thoracoscopy, Histology, Treatment Outcome

## Abstract

**Introduction:**

The objective of this study was to develop a radiofrequency ablation technique to create a homogeneous scar tissue in the atrial myocardium.

**Methods:**

In the double-blinded morphological stage of the study, the left atrial appendage was used as an anatomical model to investigate the efficacy of one experimental and two conventional techniques to create ablation lines. Then, these lines were studied by morphologists. The clinical stage involved investigation of the outcomes of the developed technique for creation of ablation lines. During thoracoscopic radiofrequency fragmentation of the left atrium, all ablation lines were created using the experimental radiofrequency technique.

**Results:**

In all histological sections of ablation lines created using the criterion of “steady decrease in the time to transmurality”, there were no intact (viable) cells, in contrast to the other two conventional methods, i.e., a homogeneous scar of the atrial wall. Investigation of clinical efficacy of this developed technique revealed recurrent atrial fibrillation only in six of 137 patients (4.4%) at median follow-up time of 36 (10; 58) months. None of the patients developed specific complications (wall perforation or bleeding). According to intracardiac mapping performed after the end of the blind period, the sources of atrial fibrillation in these six patients were outside the radiofrequency ablation zone (perimitral or in the right atrium).

**Conclusion:**

A steady decrease in the time to transmurality should be considered as the priority intraoperative criterion for the formation of a homogeneous scar during radiofrequency ablation of the left atrium wall using a bipolar ablation clamp.

## INTRODUCTION

The maze procedure is recognized as the gold standard for the treatment of patients with atrial fibrillation (AF). The idea of this procedure is to create, in an incision way, linear continuous lesions in the left and right atrial walls. This is performed to create scars, after sewing along cutting lines that limit arrhythmogenic areas of the atria. One of the key factors defining the efficacy of the maze procedure is the quality of created scars ― they lack viable cells. Such scars are called homogeneous^[1]^.

Clinical implementation of devices for radiofrequency (RF) ablation of the atrial wall has reduced the invasiveness and duration of the maze procedure. The distinctive feature of this procedure is preservation of atrial wall integrity and the isolation effect achieved due to structural lesion of the atrial wall^[2]^.

In this case, transmurality is considered as the criterion for the quality of a created ablation line. However, transmurality is not evidence for death of all cells in the affected area of the atrial wall. Preservation of live cells in the area of the scar tissue formed with the help of a RF device can cause recurrent AF^[3-5]^.

To eliminate this drawback, surgeons tend to apply many repeated RF applications to the same area of the myocardium.

In this case^[1,2,6,7]^:

- The total number of applications is not regulated and standardized.
- There are no non-invasive techniques to detect intact myocardial cells in the area of RF ablation during surgical procedure.
- Adequacy of the created ablation line can only be assessed by indirect signs because even the conduction block does not guarantee the absence of live (or hibernated) myocardial cells in the area of RF ablation.

This study is devoted to the development of a technique for creation of a homogeneous scar tissue using a bipolar RF ablation clamp and to the investigation of the clinical efficacy of the developed technique.

## METHODS

### Experimental Stage

**Fig. 1:**
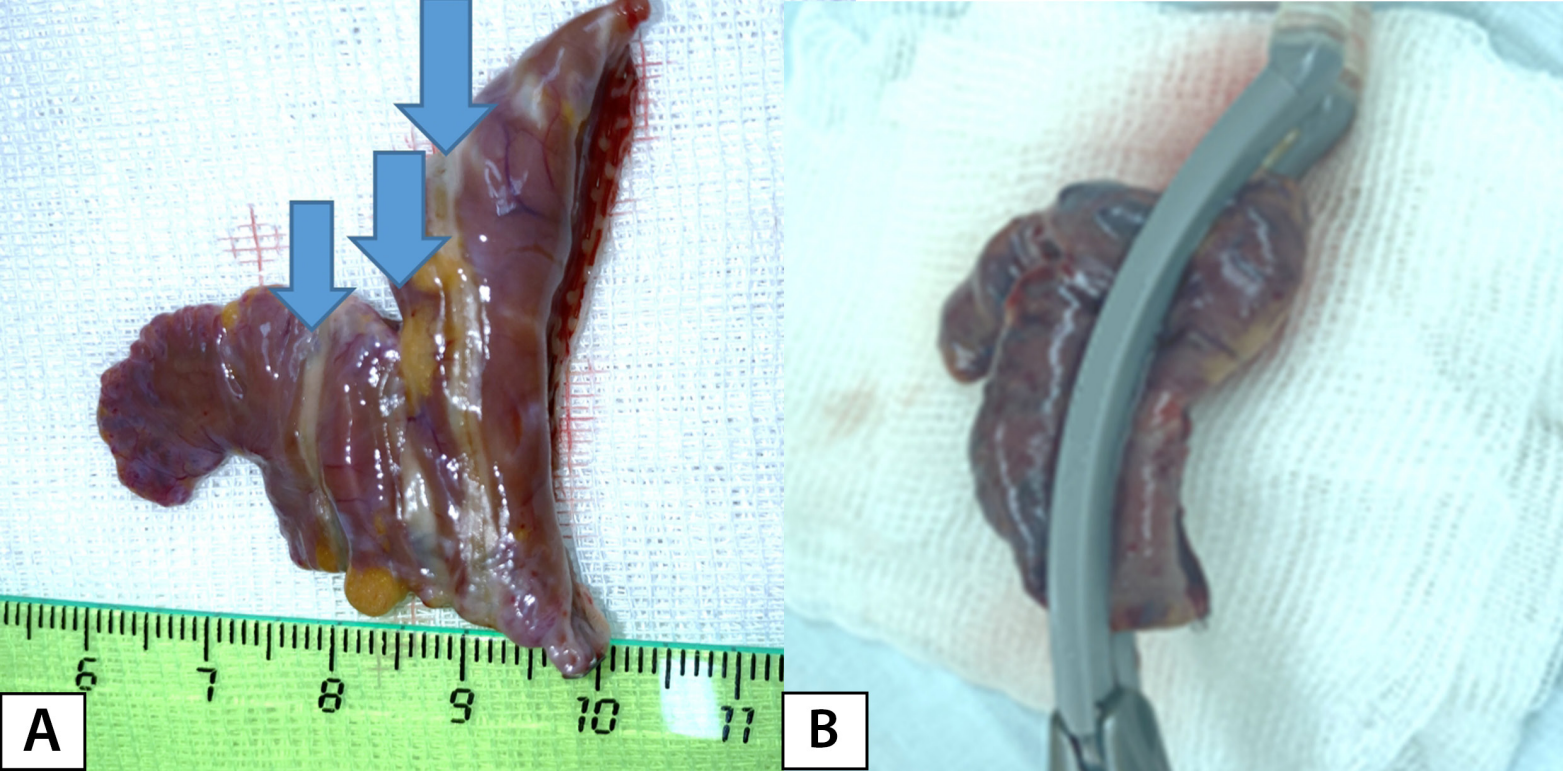
A) Ablation lines (indicated by arrows) on a resected left atrial appendage. B) Position of the left atrial appendage in an ablation clamp.

During the study, we investigated the morphology of ablation lines created by three different techniques using a Cardioblate™ Gemini™ device connected to a Cardioblate™ 68000 Surgical Ablation System Generator (Medtronic Inc, Minneapolis, Minnesota, United States of America). Ablation lines were created on the left atrial (LA) appendage resected in 18 patients whose only disease was non-paroxysmal AF. The LA appendage was resected using a stapler. In this case, blood remained in the cavity of the LA appendage. Immediately after resection, three non-intersecting ablation lines were created on each LA appendage (Figure 1A). To create each ablation line, the entire LA appendage was placed between the jaws of the ablation device (Figure 1B). Thus, an epicardial effect on duplication of the wall of the left atrium with blood in the lumen was reproduced on each LA appendage included in the study. Similarly, ablation lines were created on the LA wall during thoracoscopic RF fragmentation of the left atrium in all patients involved in this investigation.

If several RF applications were applied to create the ablation line, each subsequent application was delivered without opening the jaws of the ablation device. The duration of each application was controlled by the RF energy generator in accordance with the manufacturer’s criteria. Each individual application was delivered until the RF energy generator signaled achieving transmurality and interrupted the procedure.

The lines were created using three different techniques:

- Technique 1 (six LA appendages): a single RF application was applied to create the ablation line. The application was completed, and jaws of the RF device were unclamped when the RF energy generator signaled achieving transmurality.
- Technique 2 (six LA appendages): three RF applications were applied to create the ablation line. Each application was completed when the RF energy generator signaled achieving transmurality; however, the jaws of the ablation device were not opened between each of the applications.
- Technique 3 (six LA appendages): to create the ablation line, RF applications were applied until a steady decrease in the time to transmurality. A steady decrease in the time to transmurality was considered steady if the time was reduced by 15-20% and this was repeated at least three times in a row compared to the time achieved in the first RF application in the series. Each RF application was completed when the RF energy generator signaled achieving transmurality. The jaws of the ablation device were not opened and displaced between each of the RF applications.

A total of 54 ablation lines were created and investigated on 18 LA appendages.

Next, the LA appendage was fixed in a 10% buffered formalin solution, processed in a Thermo Scientific™ STP 120 Spin Tissue Processor, and embedded in Histomix® paraffin medium using a Tissue-Tek® TEC™ 5 modular paraffin embedding system. Sections, which were produced from paraffin blocks, were subjected to histological examination in accordance with the principles of histological techniques.

Deparaffinized sections, 3-5 µm thick, were stained with hematoxylin and eosin and embedded in BioMount™ medium. In microscopic examination, we used a NIKON DS-Fi 1 digital camera coupled to a NIKON 50s microscope and the Nis-Elements AR 4.12.00 software to produce images.

It was a double-blinded study - a morphologist who conducted histological examination of samples did not know how each specific line was created on the LA appendage. The sealed envelope with information on the technique used for creation of the ablation line was opened after obtaining the histological findings.

### Clinical Stage

The study was conducted between April 2017 and September 2021. The criteria for inclusion of patients in the study were as follows:

- Instrumentally confirmed AF.
- Failure of conservative therapy with class I and III antiarrhythmic drugs (Vaughan-Williams classification).

The criteria for exclusion of patients from the study were as follows:

- A history of surgical treatment of the chest organs.
- Cardiac conduction disorders at the time of deciding for surgical treatment.
- A history of sick sinus syndrome.
- Thrombosis of the LA appendage detected no later than one day before surgery.
- Hemodynamically significant atherosclerotic lesions of the coronary arteries and myocardial ischemia at the time of deciding for surgery.
- Valvular heart disease.
- Thyroid dysfunction at the time of surgery. A total of 137 patients were included in the study (Table 1). At the time of surgery, the only heart disease in all 137 patients was non-paroxysmal non-valvular AF. Specific instrumental examination included:

**Table 1:** Clinical characteristics of patients included in the study (N=137, 100%).

Variable	N (%)/Median (min; max)
Risk of thromboembolic complications, CHA₂DS₂-VASc score	4 (2; 6)
History of endocardial treatment aimed at restoration and long-term maintenance of sinus rhythm	21 (15%)
Obesity (body mass index ≥ 30)	57 (42%)
Atherosclerosis of peripheral (in particular, coronary) arteries	62 (45%)
Diabetes mellitus	10 (7%)
Hypertension (systolic blood pressure ≥ 140 mmHg)	92 (67%)
History of amiodarone-induced complications	19 (14%)
Left ventricular ejection fraction (according to Simpson)	57 (38; 67)
Anteroposterior size of the left atrium, mm	45 (33; 70)
Left atrial volume, ml	86 (39; 175)
Indexed left atrial volume, ml/m^2^	42 (18; 89)

CHA₂DS₂-VASc=congestive heart failure, hypertension, age ≥ 75 years (doubled), diabetes, stroke (doubled), vascular disease, age 65 to 74 years, and sex category (female)
- Transthoracic echocardiography that was performed in the preoperative and late postoperative periods.
- Transesophageal echocardiography that was performed in the preoperative and late postoperative periods.
- Angiography of the coronary arteries.
- 24-hour and 72-hour electrocardiogram monitoring that was performed in the preoperative and late postoperative periods.

In the postoperative period (three, nine, and 12 months, then every 12 months or upon recurrence of complaints associated with AF), patients visited the hospital for their comprehensive follow-up examination - 24-hour or 72-hour electrocardiogram monitoring and transthoracic echocardiography. When taking medical history in the postoperative period, attention was paid to recurrence of AF (instrumental examination), subjective patient assessment of their condition (as an indication for further instrumental examination), adherence of the patient to the prescribed therapy, duration of taking the prescribed drugs (antihypertensive, anticoagulant, and antiarrhythmic medications, beta-blockers), and indications for pacemaker implantation. In the case of an instrumentally registered recurrence of AF, the patient underwent endocardial mapping.

### Surgery

A prerequisite for the surgical procedure is using a double-lumen tube for separate ventilation of the lungs.

Surgical access to the heart was performed through trocars. Firstly, three ports were placed in the right hemithorax - two along the anterior axillary line in the 3^rd^ and 6^th^ intercostal spaces and one, for the camera, in the 4^th^ intercostal space.

The pericardium was opened anteriorly and parallel to the phrenic nerve. The length of the pericardial incision was determined by the goal to achieve a clear visualization of the pulmonary venous collector and the superior and inferior vena cava.

The duplication of the epicardium posteriorly to the superior and inferior vena cava was fenestrated. Then, the guides for the ablation device were placed into the transverse and oblique sinuses of the pericardium through a trocar in the 6^th^ intercostal space.

After that, three ports were installed in the left half of the chest - one along the anterior axillary line in the 3^rd^ intercostal space, one along the midaxillary line in the 4^th^ intercostal space, and one along the line between the anterior and median axillary lines in the 6^th^ intercostal space.

The pericardium was opened posteriorly and parallel to the phrenic nerve. Pericardiotomy was performed directly over the root of the lung. The length of the pericardial incision was determined by the goal to achieve a clear visualization of the left atrial appendage and the pulmonary venous collector.

Then, the guides for the ablation device were extracted from sinuses of pericardium into the left pleural cavity. Through the port in the 6th intercostal space, both guides were taken out, and an ablation device was connected to them.

The only RF type of surgical device that was used during procedure in all 137 patients was the Cardioblate™ Gemini™-S bipolar clamp (Medtronic, Minneapolis, Minnesota, United States of America).

The ablation device was first inserted into the pericardial cavity with an upward curvature relative to the patient’s spine. Under control of hemodynamics, the clamp was closed around the wall of the left atrium. Then, the ablation procedure was started. The time required to achieve transmurality as a result of the first application was fixed. Then, without opening and without displacement of the clamp, a row of ablation applications was performed until the steady decrease in the time to achieve transmurality.

The clamp was opened and removed from the pericardial and pleural cavities. After reconnection to the guides, the ablation device was reintroduced into the pericardial cavity. This time the ablation device was inserted into the pericardial cavity with a downward curvature relative to the patient’s spine. Ablation procedure was repeated in the same manner. After achieving the steady decrease in the time to achieve transmurality, the clamp was opened and extracted from the pericardial and pleural cavities.

The left atrial appendage was resected with a Medtronic Echelon™ (Medtronic, Minneapolis, Minnesota, United States of America) stapler and a “60+” cassette with 3.6-mm high staples. The ligament of Marshall was destroyed using coagulation or LigaSure™ Maryland Jaw Thoracic Sealer/Divider Echelon (Medtronic, Minneapolis, Minnesota, United States of America).

After that, the left pleural cavity was drained. Then, the procedure of ablation of the left atrial wall was started from the side of the right pleural cavity. The sequence and method of ablation impact on the right half of the left atrium did not differ from that one on the left half of the left atrium.

Finally, bilateral epicardial stimulation was performed to check the block of conduction. According to bilateral epicardial stimulation, the procedure was proved effective in all 137 patients.

All 137 patients underwent thoracoscopic RF fragmentation of the LA^[8,9]^; and all underwent surgery using the developed technology for creation of a homogeneous scar tissue.

The median time of total duration of the procedure was 133 (93; 148) minutes. The median time of total time of ablation during procedure was 38 (24; 55) minutes.

So, the technology for creation of a homogeneous scar tissue using RF ablation of the atria was as follows:

1. The ablation line was created by consecutive repeated RF applications to the same anatomical area of the atrium using a bipolar ablation clamp, without opening and displacement of the clamp jaws.
2. The ablation line was considered created only after a steady decrease in the time to transmurality, which was the intraoperative criterion for the quality of the created ablation line.
3. The criterion of a steady decrease in the time to transmurality was defined as a 15-20% reduction in the time to transmurality, repeated at least three times in a row, compared to the time achieved in the first RF application in the series (Figures 2 A and B).
  **Fig. 2:**
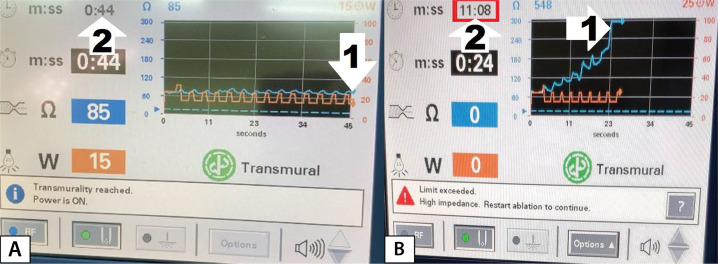
A decrease in the time to transmurality during radiofrequency (RF) ablation of the left atrial wall. A) First RF application in the series. B) Final RF application. 1 - achievement of transmurality; 2 - total duration of RF application from the time of the first RF application: the steady decrease in the time to achieve transmurality is obtained.

### Ethical Review

The study was performed in accordance with the principles of the Helsinki Declaration. The study protocol was approved by the local ethics committee (No. 18 of December 27, 2016). All patients gave informed consent to participate in the study and to use anonymized data on their health status in the preoperative and postoperative periods for scientific purposes.

### Statistical Analysis

The study was performed in accordance with the principles of evidence-based medicine. Statistical data processing was performed using the IBM Corp. Released 2015, SPSS Statistics for Windows, version 23, Armonk, NY: IBM Corp. Qualitative clinical and demographic characteristics (categorical variables) are presented as frequencies (N) and percentages (%). Quantitative clinical and demographic characteristics (continuous variables) are presented as the median with the minimum and maximum range indicated in parentheses - Me (min; max). The dependence of the time to a clinically significant event on the time of its onset was analyzed using the Kaplan-Meier method.

## RESULTS

### Experimental Stage

The histological structure of ablation lines created on the myocardium of the LA appendage was investigated.

We studied a total of 900 histological sections produced by ablation of 18 specimens (three ablation lines per each LA appendage).

**Fig. 3:**
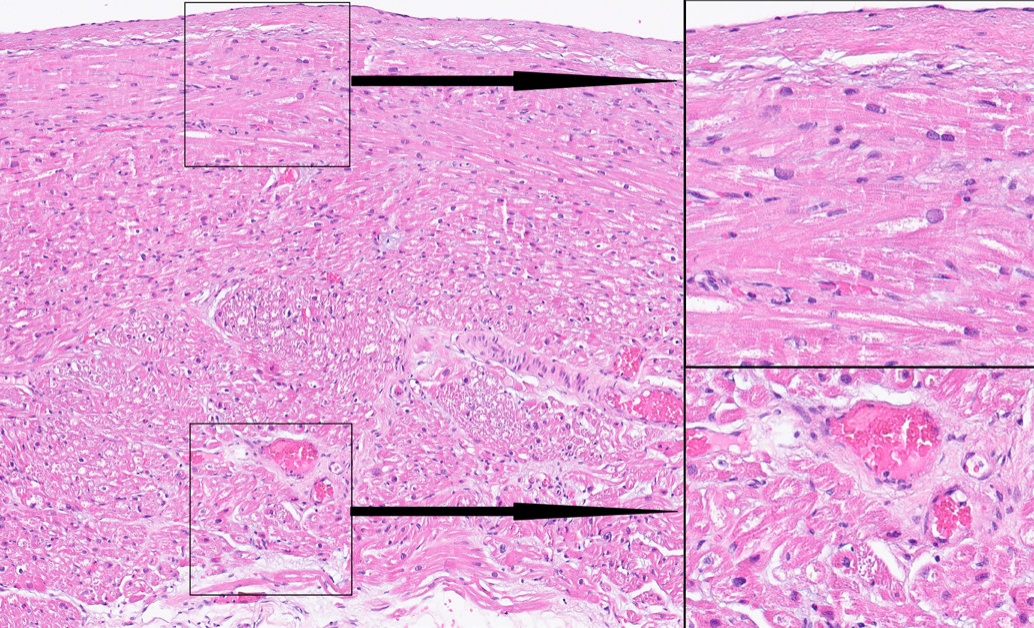
Technique 1 - standard technology for ablation line creation. Histological examination of ablation lines. Incomplete ablation line: subpericardial cells were damaged; subendocardial myocardiocytes remained intact. Micrograph. Staining with hematoxylin and eosin. Magnification: ×125 (left side of the figure); ×1000 (right side of the figure).

Investigation of 300 histological sections of ablation lines created by technique 1 revealed that the damaged cells were located subepicardially and accounted for 1/3-1/2 of the atrial wall thickness. In this case, subendocardial myocardiocytes remained intact (Figure 3).

**Fig. 4:**
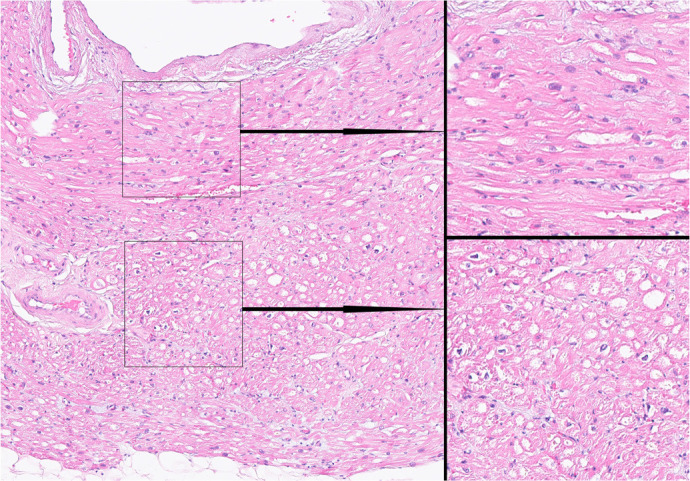
Technique 2. Histological examination of ablation lines. Incomplete ablation line: subepicardial cells and cells of the middle layer of the atrial wall were damaged; subendocardial myocardiocytes remained intact. Micrograph. Staining with hematoxylin and eosin. Magnification: ×125 (left side of the figure); ×1000 (right side of the figure).

Investigation of 300 histological sections of ablation lines created by technique 2 showed that the damaged cells were located subepicardially and accounted for 2/3-3/4 of the atrial wall thickness, with the subendocardial myocardial layers remaining intact (Figure 4).

**Fig. 5:**
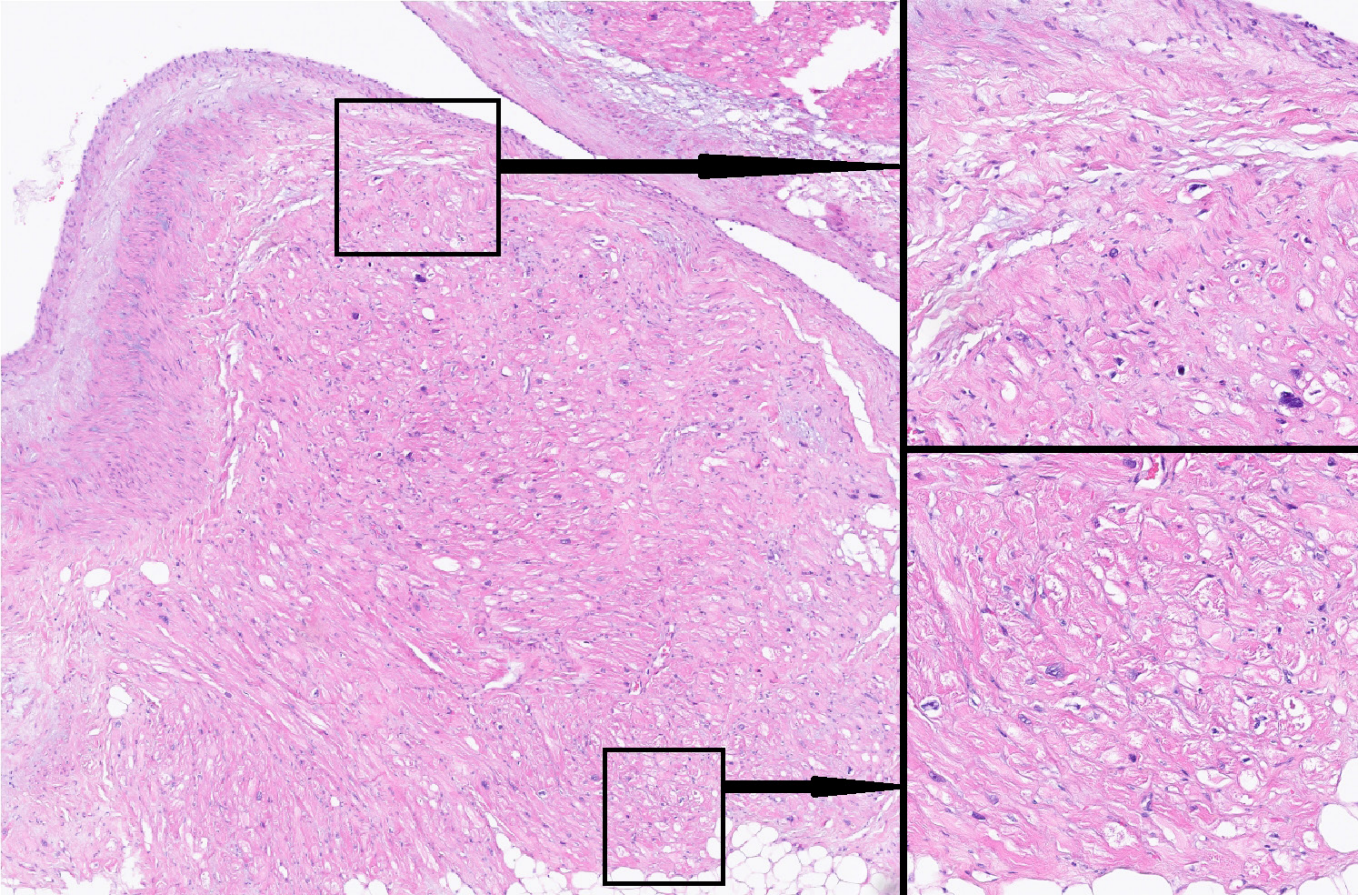
Technique 3 - new technology used for homogeneous scar tissue creation. Histological examination of ablation lines. Complete ablation line, no intact (viable) cells within the created lesion. Micrograph. Staining with hematoxylin and eosin. Magnification ×125 (left side of the figure); ×1000 (right side of the figure).

Investigation of 300 histological sections of ablation lines created by technique 3 revealed no intact (viable) cells within the created lesion. The result is shown in Figure 5.

### Clinical Stage

None of the patients included in the study developed complications associated with a change in the ablation procedure (atrial wall perforation, bleeding). All 137 (100%) patients were restored to sinus rhythm after completion of ablation treatment.

During the blind period, all patients took amiodarone according to the regimen recommended by the manufacturer, beta-blockers under close heart rate monitoring, and anticoagulants. In the case of contraindications to amiodarone, sotalol was prescribed (N=19, 14%) at a dose of at least 80 mg twice a day. The dose might be increased depending on individual tolerance. By the end of the blind period, all patients had a stable sinus rhythm. After the end of the blind period, antiarrhythmic drugs (amiodarone, sotalol) were terminated.

**Fig. 6:**
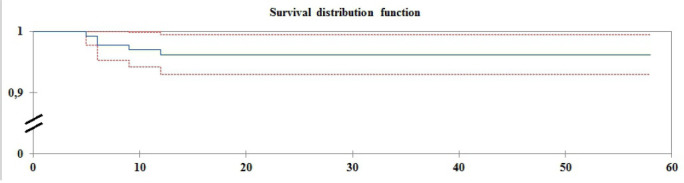
Analysis of freedom from recurrent atrial fibrillation in the late postoperative period; median follow-up time of 36 (10; 58) months.

In the late postoperative period - median follow-up time of 36 (10; 58) months -, six patients (4.4%) returned to the baseline condition (Figure 6).

**Fig. 7:**
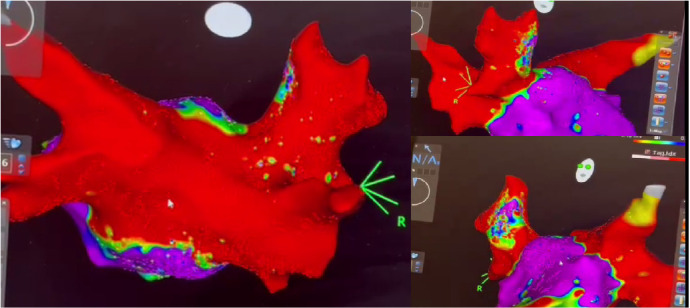
Findings of endocardial electrophysiological examination in a patient with recurrent atrial fibrillation. Electrical isolation of the pulmonary veins and left atrial posterior wall (marked by red).

Endocardial examination in all six patients with recurrent AF confirmed electrical isolation of the pulmonary veins and LA posterior wall. Recurrent AF developed because of extrapulmonary triggers (Figure 7).

## DISCUSSION

Obviously, only surgical techniques may be relatively radical in long-term maintenance of sinus rhythm after stopping AF. In this case, the most aggressive operation, the maze procedure, remains the most radical treatment among all surgeries^[10-12]^.

The classic maze procedure and its two modified versions developed by the inventor, maze II and maze III, involve sequential cutting and sewing of the right and LA walls^[13]^. This results in mechanical destruction of re-entry wave pathways and mechanical isolation of pathological impulse conduction pathways^[1,14]^.

Devices for RF ablation of the atrial wall have made the maze procedure faster, easier, and more widespread. However, the clinical outcome of the RF modification of the operation is worse than the outcome of the classic maze procedure^[15,16]^.

One of the causes for better outcomes of the classic maze procedure is the creation of a homogeneous scar tissue in the atrial wall, which is due to mechanical disturbance of atrial wall integrity ― cutting followed by sewing^[17]^. RF ablation of the atria results in tissue damage, with continuity of the atrial wall being preserved. In this case, the generally accepted criterion for the quality of linear RF lesions formed in the atrial wall is transmurality, that is a physical parameter based on a change in tissue resistance to an impulse in the ablation area due to penetration of RF energy through all layers of the atrial wall. However, despite the achieved transmurality, intact cells may remain within the ablation line. Thus, achieving transmurality does not always guarantee the formation of a homogeneous scar tissue^[18,19]^.

Surgical techniques using RF devices are characterized by a diversity not only in the target scheme comprising a different number of ablation lines but also in the number of RF applications applied to create each individual line.

The study revealed that the criterion for the quality of the created ablation line is a steady decrease in the time for achieving transmurality produced with a series of RF applications to the same area of the LA myocardium, provided that the applications are applied without opening and displacement of the ablation clamp jaws. The created ablation line is free of intact cells of the atrial wall. Therefore, this ablation line may be considered complete because the scar tissue formed at this site is homogeneous (does not contain intact cells of the atrial wall).

The validity of the developed technique for creation of ablation lines by RF ablation of the atrial wall is confirmed by the clinical outcome of thoracoscopic epicardial RF ablation of the atrial wall: after the end of the blind period, freedom from all forms of AF in patients who underwent thoracoscopic RF fragmentation of the left atrium was 95.6% at median follow-up time of 36 (10; 58) months.

## CONCLUSION

A steady decrease in the time to transmurality should be considered as the priority intraoperative criterion for the formation of a homogeneous scar tissue during ablation lines creation by RF ablation of the left atrium using a bipolar ablation clamp. Application of this criterion upon thoracoscopic RF fragmentation of the left atrium provided freedom from recurrent AF in 131 out of 137 patients (95.6%) at median follow-up time of 36 (10; 58) months, without increasing the number of specific complications.
